# Supplementary material for: The Road More Travelled: The Differential Effects of Spatial Experience in Young and Elderly Participants
Source: Int J Environ Res Public Health. 2021 Jan 15;18(2):709. doi: 10.3390/ijerph18020709 (PMC7830856; doi:10.3390/ijerph18020709)
Supplement: Supplementary file 1 [file ijerph-18-00709-s001.pdf]

Table S1. Correlation Matrix.

	§*AGE*	*GENDER*	*EDUCATION*	*COGNITIVE FUNCTIONING*	*RESIDENTIAL LOCATION*	*INCOME*	*MODE OF EXPLORATION*	*FREQUENCY OF EXPLORATION*	*COORDINATE*	*CATEGORY*
AGE	1									
GENDER	-0.010	1								
EDUCATION	0.532***	0.073	1							
COGNITIVE FUNCTIONING	0.680***	0,101	0.573***	1						
RESIDENTIAL LOCATION	-0.063	-0.027	-0.152**	-0.062	1					
INCOME	0.137**	-0.024	0.155**	0.106*	-0.523***	1				
MODE OF EXPLORATION	0.045	-0.045	0.034	0.042	0.072	-0.017	1			
FREQUENCY OF EXPLORATION	0.145**	-0.115*	0.288**	0.190**	0.039	-0.013	0.130**	1		
COORDINATE	0.362**	0.084	0.386**	0.379**	0.043	0.013	0.092	0.553***	1	
CATEGORY	0.275**	-0.075	0.298***	0.277***	0.063	0.014	0.072	0.572***	0.872***	1

Note: * $p < .05$, ** $p < .01$, *** $p < .001$; § the variable Age is encoded as follows: young=1, elderly=-1.

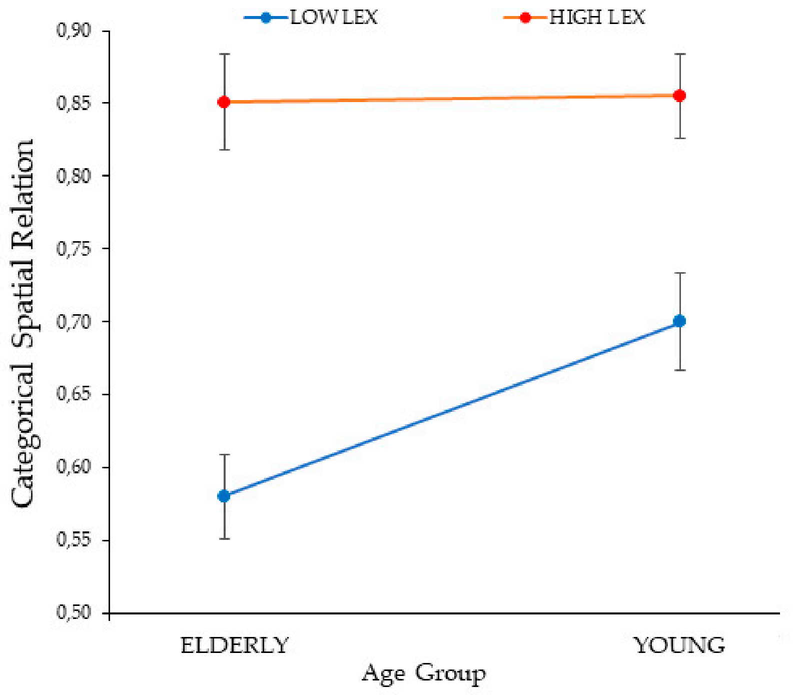

Figure S1. A line graph depicting the interaction effect between the Level of Experience Index (LEX) and Age Group on categorical spatial relation (Model 1).

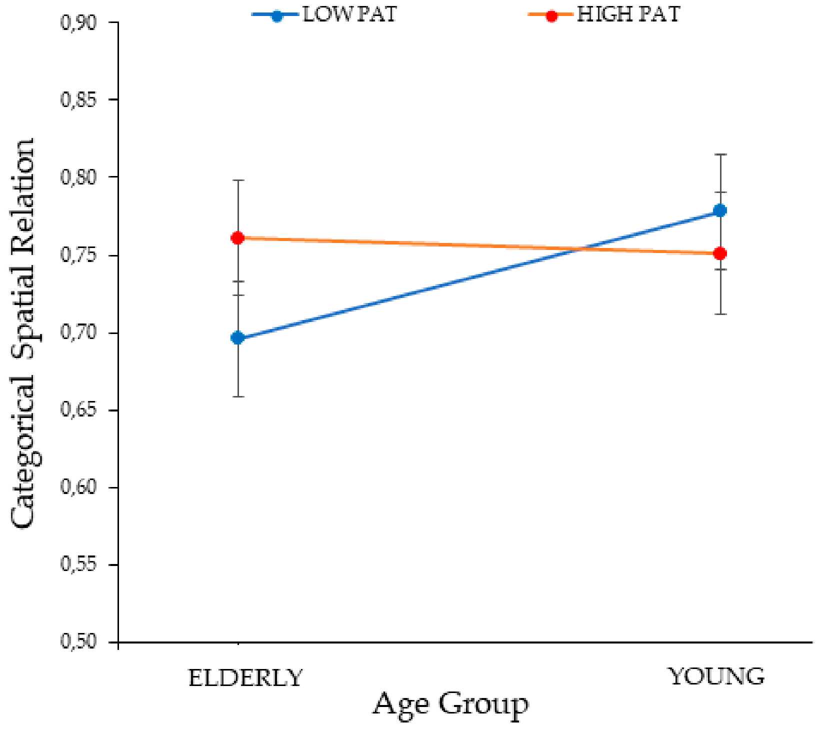

Figure 2. A line graph depicting the interaction effect between the Passive Active Transportation index (PAT) and Age Group on categorical spatial relation (Model 2).

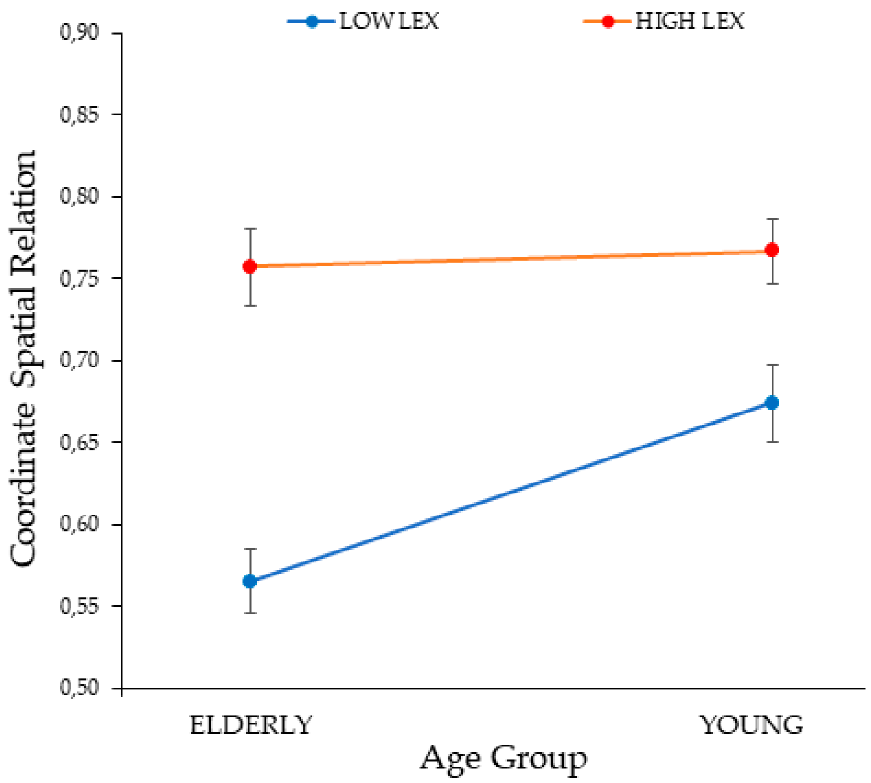

Figure S3. A line graph depicting the interaction effect between the Level of Experience Index (LEX) and Age Group on coordinate spatial relation (Model 3).

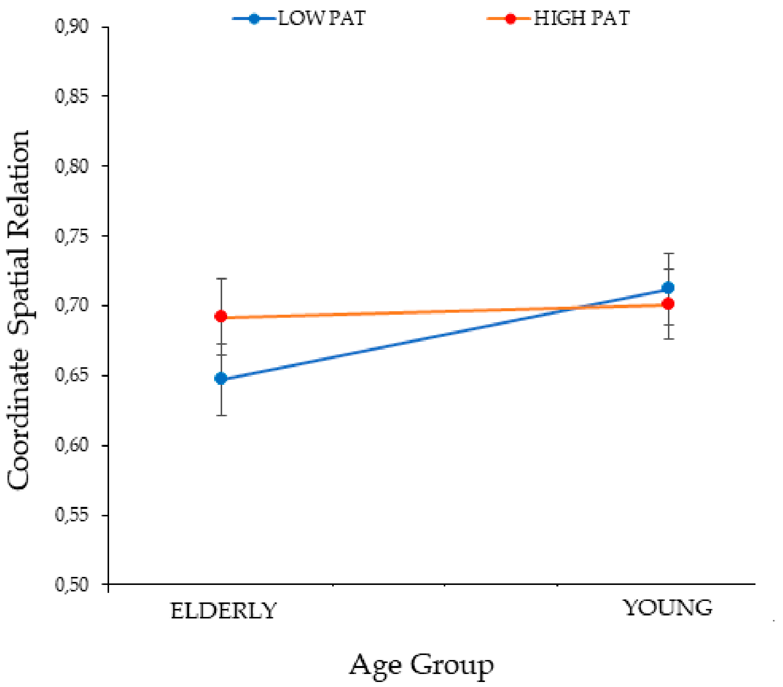

Figure S4. A line graph depicting the interaction effect between the Passive Active Transportation index (PAT) and Age Group on coordinate spatial relation (Model 4).
